# Deep learning black box and pattern recognition analysis using Guided Grad-CAM for phytolith identification

**DOI:** 10.1093/aob/mcaf088

**Published:** 2025-05-30

**Authors:** Iban Berganzo-Besga, Hector A Orengo, Felipe Lumbreras, Monica N Ramsey

**Affiliations:** Computational Social Sciences and Humanities Department, Barcelona Supercomputing Center (BSC-CNS), Barcelona 08034, Spain; Ramsey Laboratory for Environmental Archaeology (RLEA), Department of Anthropology, University of Toronto Mississauga (UTM), Mississauga, ON L5L 1C6, Canada; Landscape Archaeology Research Group (GIAP), Catalan Institute of Classical Archaeology (ICAC), Tarragona 43003, Spain; Computational Social Sciences and Humanities Department, Barcelona Supercomputing Center (BSC-CNS), Barcelona 08034, Spain; Landscape Archaeology Research Group (GIAP), Catalan Institute of Classical Archaeology (ICAC), Tarragona 43003, Spain; Catalan Institution for Research and Advanced Studies (ICREA), Barcelona 08010, Spain; Computer Vision Center (CVC), Bellaterra (Cerdanyola del Vallès) 08193, Spain; Department of Computer Science, Universitat Autònoma de Barcelona (UAB), Bellaterra (Cerdanyola del Vallès) 08193, Spain; Ramsey Laboratory for Environmental Archaeology (RLEA), Department of Anthropology, University of Toronto Mississauga (UTM), Mississauga, ON L5L 1C6, Canada

**Keywords:** Computational archaeology, phytolith, deep learning, black box, pattern recognition, visual explainer, best practice, *Avena sativa*, *Hordeum spontaneum*, *Triticum boeoticum*, *Triticum dicoccoides*

## Abstract

**Background and Aims:**

In this article, visual explainers are applied to give transparency to the black box of a trained VGG19 model for the identification of multi-cell phytoliths of the *Avena*, *Hordeum* and *Triticum* genera. The aim is to demonstrate its proper learning by visually highlighting the phytolith characteristics that the deep learning model uses to classify these phytoliths; we then compare the model’s methods with those employed manually by archaeobotanists.

**Methods:**

The visual explainers used for this purpose are Grad-CAM, Guided Backpropagation and Guided Grad-CAM, the last being a combination of the previous two. This combined tool not only highlights the most relevant regions when classifying phytoliths on microscope images, but also emphasizes every detail within those areas.

**Key Results:**

The importance of the wave pattern as a decision-maker (key identifying characteristic) when classifying phytoliths has been demonstrated for 91 % of the microscope images. Similarly, the papillae have been a key in 86 % of *Avena* images, in 94 % when images included papillae, and the dendritic long-cell shape in 38 % of *Triticum* images.

**Conclusions:**

The analysis of the microscope images using Guided Grad-CAM has validated the established patterns in phytolith identification, such as highlighting the significance of the wave pattern. Additionally, it revealed that varying phytolith characteristics might be prominent for different genera and led to the discovery that dendritic long-cell shape, as an independent category, is also distinctive. This research is part of an effort to establish a set of computer vision best practices in computational archaeology.

## INTRODUCTION

### Machine learning

As a subset of artificial intelligence (AI), machine learning (ML) involves algorithms that learn, through a training process, patterns from data without needing explicit step-by-step programming (Samuel, 1959; Jordan and Mitchell, 2015). Deep learning (DL) is a specialized area within ML that uses neural networks to learn complex patterns directly from data, eliminating the need for manual feature extraction common in traditional ML approaches (Rumelhart *et al*., 1986; LeCun *et al*., 2015). Convolutional neural networks (CNNs) are a type of DL algorithm especially effective in image-related tasks because they use convolutional filters, mathematical matrix operations that help enhance and detect certain features such as textures or edges in images (LeCun *et al*., 1998; Krizhevsky *et al*., 2012). VGG19, developed by researchers in the University of Oxford’s Visual Geometry Group (VGG), is an example of such a CNN with 19 layers. The main contribution of this architecture to previous ones is the increase in convolutional network depth thanks to the use of small convolutional filters, improving in this way the performance of previous approaches (Simonyan and Zisserman, 2015). This VGG19 algorithm has been widely used in various image classification tasks, including identifying plant species (Siddharth *et al*., 2022; Rohith and Bhuvaneswari, 2023).

### Related work

In a previous article (Berganzo-Besga *et al*., 2022), we successfully developed a DL model, a trained VGG19 algorithm, for the detection and classification of multi-cell phytoliths, specifically three key grass husk multi-cell types: wheat (wild einkorn, *Triticum boeoticum*; wild emmer, *Triticum dicoccoides*), barley (wild, *Hordeum spontaneum*) and oat (domestic common oat, *Avena sativa*). These three classes, *Avena*, *Hordeum* and *Triticum*, are important phytoliths for the study of agricultural origins in Near East archaeology.

**Fig. 1.**
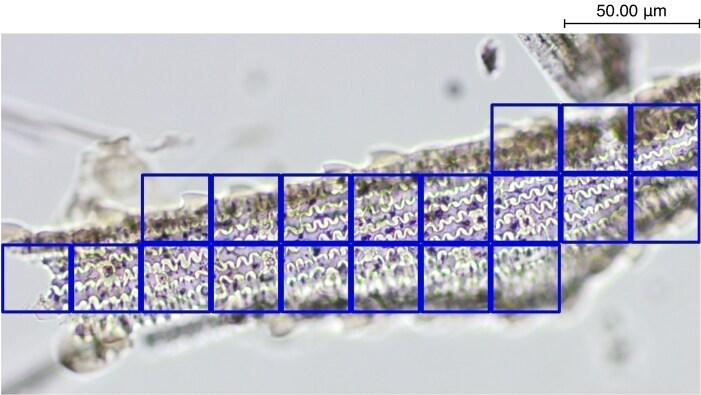
*Triticum* phytolith image from the original article (Berganzo-Besga *et al*., 2022) correctly classified. See the original article (Berganzo-Besga *et al*., 2022) for interpretation.

This trained algorithm achieved a classification accuracy of 93.68 %, being 98.44 % in *Avena*, 93.55 % in *Hordeum* and 89.06 % in *Triticum* (Fig. 1). However, the absence of any kind of visual explainer (depictive representation of AI model focus via attention heat maps) not only prevents us from knowing the character (morphological features of the phytoliths) that the AI learnt and follows to classify these phytoliths, but even whether the said classification is carried out correctly despite the high accuracy metrics. The use of ML algorithms to classify phytoliths is a rapidly developing research area (Berganzo-Besga *et al*., 2022; Andriopoulou *et al*., 2023; Díez-Pastor *et al*., 2024), and ensuring the integrity of this fast-moving research through the use of visual explainers is critical for the wider acceptance of these models in archaeology.

### Hidden patterns

**Fig. 2.**
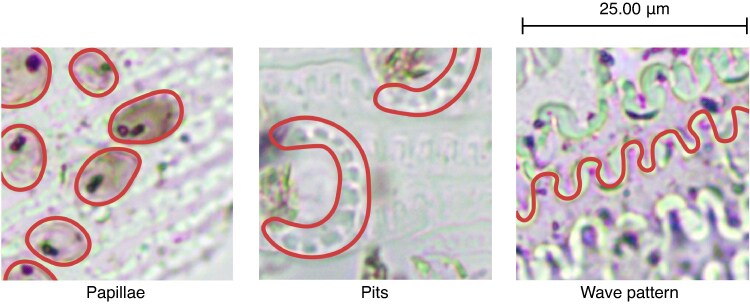
Near East husk multi-cell phytolith characters (classification patterns) outlined by Rosen (1992) in various 256-px crop example images: papillae, pits and wave pattern (marked on the images).

As mentioned in our previous article (Berganzo-Besga *et al*., 2022), the method for identifying Near East husk multi-cells was first detailed by Rosen (1992), where the characteristics of wheat, barley, oat and rye grass were outlined based on (1) papilla size, shape and regularity, (2) the number of pits around the papillae, and (3) the wave pattern, the negative space between dendritic long cells (Fig. 2). This approach is still in use for the identification of these Near Eastern grasses.

Deep learning is not only a way to automate complex processes (LeCun *et al*., 2015; Berganzo-Besga *et al*., 2023) but it also has the potential to find hidden patterns by explaining their learning and decision-making process (Hassija *et al*., 2023), which improves the comprehension of its predictions. These patterns learnt by the algorithms can be visualized through a series of visual explainers (Buhrmester *et al*., 2021), which provide us with information on how the classification has been carried out. These visual explainers are a visual interpretation to identify the most discriminative area in an image, clarifying the decision made by the algorithm. Applying these tools to our phytolith identification DL model can give us an idea of what the algorithm has focused on to classify the phytoliths as genus *Avena*, *Hordeum* or *Triticum*.

Thus, we can determine whether the AI uses the same patterns as those indicated by Rosen (1992), whether it uses only some of them, or whether it uses different ones, some possible hidden patterns. In this way we can better understand and improve the method of phytolith identification.

### Black box issue

**Fig. 3.**
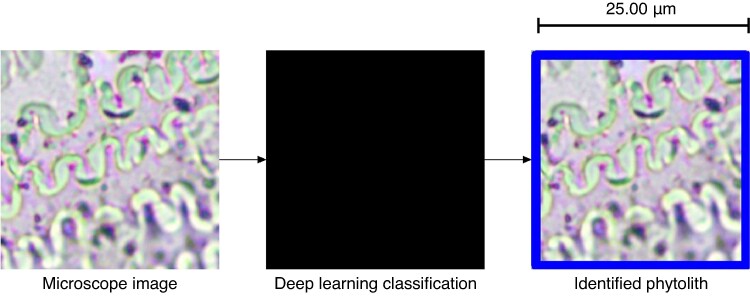
Explanatory image of a DL black box for phytolith classification using the algorithm of Berganzo-Besga *et al*. (2022). The original 256-pix crop example image is classified as *Triticum*. See the original article (Berganzo-Besga *et al*., 2022) for interpretation.

In recent years, DL has experienced a peak thanks to technological improvements (Krizhevsky *et al*., 2012; Goodfellow *et al*., 2014; Vaswani *et al*., 2017), which has led to an upsurge of these algorithms in the field of archaeology (Zingman *et al*., 2016; Fetaya *et al*., 2020; Orengo *et al*., 2021), such as the identification of phytoliths (Berganzo-Besga *et al*., 2022), but also some doubts about their reliability (Hassija *et al*., 2023). One of the known problems within DL is the black box issue (Ali *et al*., 2023), i.e. it is unknown how a DL algorithm classifies (Fig. 3). This issue is the reason why the mentioned visual explainers (Buhrmester *et al*., 2021) are not only necessary to understand patterns, but also to ensure that these patterns are accurate.

There is an urban legend, known as The Neural Net Tank Urban Legend (Branwen, 2011; Jackson, 2019), that explains that accurate results do not always indicate a well-trained algorithm. Next, to explain the black box issue, we will recreate the core of this legend using an algorithm trained by us to distinguish between images of a specific cat and a specific dog.

**Fig. 4.**
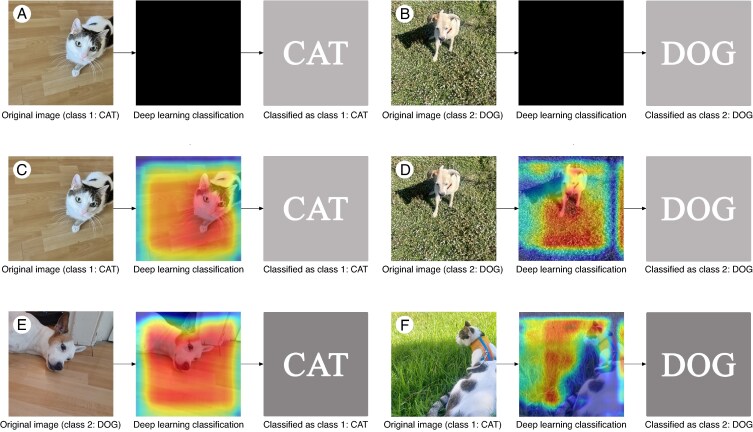
A poorly trained cat vs dog recognition model. (A) Black box issue illustrated for a first cat image example. (B) Black box issue illustrated for a first dog image example. (C) Grad-CAM applied to the first cat image example. (D) Grad-CAM applied to the first dog image example. (E) Grad-CAM applied to a second dog image example. (F) Grad-CAM applied to a second cat image example. The model correctly classifies the first image examples (C and D) and incorrectly the second ones (E and F).

We trained a VGG19 algorithm (without transfer learning) to classify into cat or dog. After 25 epochs (an epoch being one complete pass through all the training images during the learning process), we validated the model with the validation images, obtaining an accuracy of 100 % and a loss (error measurement) of 0.006, results for which we consider the trained algorithm valid. In the testing dataset we also obtain an accuracy of 100 %. As explained in Fig. 4, this high accuracy can give us the wrong impression that the algorithm works perfectly. To understand this issue first we need to analyse its black box (Fig. 4A, B). Using the visual explainer known as Gradient-Weighted Class Activation Mapping (Grad-CAM) (Selvaraju *et al*., 2017) we can observe (checking the yellowish to reddish areas of the resulting image) that the model is not classifying the images by whether they contain a cat or a dog but by their background (Fig. 4C, D). Since all the cat images were taken indoors and all the dog images outdoors, the algorithm used the background (wood vs grass) to classify them. That is why if we try to classify an image of a dog in a wooden indoor space, the model classifies it as cat, and if we try to classify an image of a cat in a grass outdoor space, it classifies it as a dog (Fig. 4E, F). As with the cat vs dog example, we need to focus not only on the appropriate source, but also on how the data are acquired to ensure there is no bias at the time. Therefore, we cannot confirm that high accuracy metrics obtained by the application of a computer vision algorithm correspond to a correct classification, without the use of visual explainers. This also serves to illustrate the importance of using data augmentation techniques to avoid undesired learning by algorithms, such as the use of brightness and colour jittering (Berganzo-Besga *et al*., 2022), and the so-called Doppelgänger technique (Berganzo-Besga *et al*., 2023).

### Article objectives

Through the application of visual explainers to our trained model we will (1) aim to find the patterns learnt by the algorithm and compare them with those established by the archaeological community (Rosen, 1992), and in doing so we can (2) guarantee that our model was trained correctly, confirming its high metrics when classifying these three classes of phytoliths.

Some visual explainers have been used in archaeology (Winterbottom *et al*., 2022), but mostly Grad-CAM (Selvaraju *et al*., 2017) applied to the classification of coins (Anwar *et al*., 2021), potsherds (Pawlowicz and Downum, 2021) and bronzes (Zhou *et al*., 2023). To achieve these objectives, we seek to use not a single visual explainer but a combination of them to improve the analysis and conclusions, as is the case with Guided Grad-CAM (Selvaraju *et al*., 2017).

## MATERIALS AND METHODS

### General workflow

To achieve these objectives, we will employ an AI-based visual approach rather than traditional manual inspection methods. Specifically, we will apply various visual explainers (as described below in the Visual explainers section) to an already trained DL algorithm (further discussed in the Deep learning model section). This process will be applied to each image in our multi-cell phytolith dataset used for our classification model (detailed in the Dataset section). The goal is to identify, by analysing these resulting images, the characteristics learnt by our model during classification.

Inspecting the highlighted areas by these visualization tools, we can determine which aspects of the images most impact the model’s classification decisions. This quantitative analysis will reveal the key characteristics that the AI model uses for classifying these multi-cell phytoliths and help us assess whether the algorithm has learnt successfully. Ultimately, this AI-based visual analysis approach will deepen our understanding of multi-cell phytolith genera and ensure our DL model is performing as intended.

### Deep learning model

**Fig. 5.**
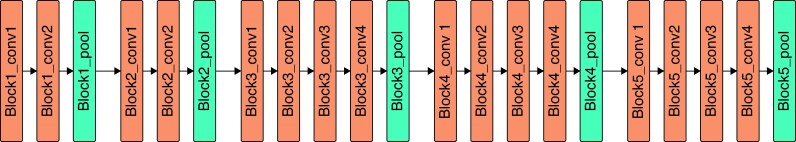
The VGG19 16 convolutional layers grouped into five blocks, each followed by a pooling layer. In addition to these 16, the VGG19 architecture also has three fully connected layers.

CNN architectures, such as VGG19, are designed with several key components (Krizhevsky *et al*., 2012; Simonyan and Zisserman, 2015): an input layer, which receives image data (image pixels), convolutional layers (which process visual features) (Fig. 5), fully connected layers (for higher-level pattern recognition), and a final dense layer, also a fully connected layer, which outputs class probabilities. As in the cat vs dog recognition example above, the idea of implementing DL is to develop a trained algorithm to classify images into the desired classes (e.g. ‘dog’ or ‘cat’). In VGG19 (Simonyan and Zisserman, 2015), each of the first two fully connected layers contains 4096 neurons, which are basic units responsible for mathematically processing inputs and generating outputs. The activation function inside the layers (Dubey *et al*., 2022), such as the rectified linear unit (ReLU) used in VGG19, is essential because it introduces non-linearity into the model, allowing it to learn complex patterns from data. This function determines whether a neuron’s output contributes to learning after convolutional and fully connected layers. Likewise, neuron weights are parameters that control how much influence each input has on the output, while gradients guide adjustments to these weights during training, helping the network improve its predictions over time (LeCun *et al*., 1998). The final dense layer, with as many neurons as classes it wants to predict, produces probabilities for each class using an activation function, representing likelihoods for each image of belonging to each category. In VGG19 (Simonyan and Zisserman, 2015), this last activation function is *softmax*, a multi-class activation function (instead of ReLU as in the rest of the layers). Our multi-cell phytolith classification model (Berganzo-Besga *et al*., 2022) is based on this VGG19 architecture and classifies images into three classes: *Avena*, *Hordeum* or *Triticum* genera.

When we use a fully connected layer in a DL CNN model, we lose the spatial information that is retained by the convolutional (*conv*) layers (Fan *et al*., 2021), which extract the characteristics of the feature to be classified or detected (e.g. ‘dog’ or ‘cat’). Likewise, the pooling (*pool*) layers, associated with the convolutional layers, guarantee greater robustness of the model against small variations in the input. Therefore, the *block5_pool* layer will be our object of study using different visual explainers to analyse how the DL model carries out the classification of the features, in our case phytoliths.

### Visual explainers

If we looked directly at the *block5_pool* layer, we would see the regions detected by the convolutional filters, highlighting textures, edges and patterns. However, this does not indicate which of these regions were crucial for the final decision. Visual explainers, like Grad-CAM in the previous cat vs dog recognition example, improves this by using the gradients from the fully connected layers. The gradients show which patterns were most important for the final decision (Selvaraju *et al*., 2017). This helps distinguish only the areas that truly influenced the classification. This approach allows us to understand not just what the network detects, but what it has truly learnt to prioritize for making predictions (class identification).

**Fig. 6.**
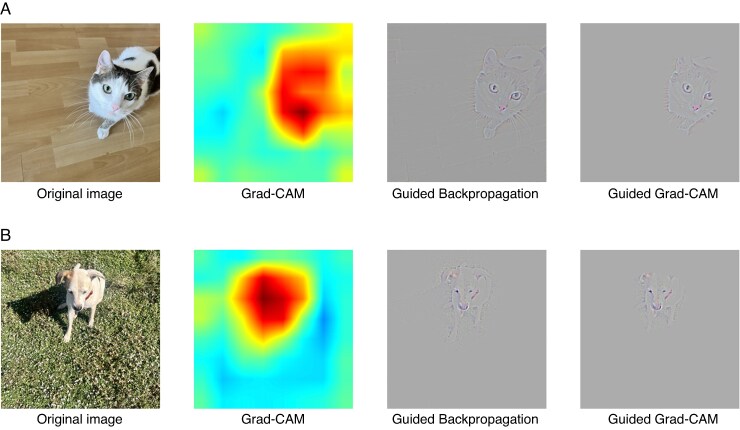
Visual explainers (Grad-CAM, Guided Backpropagation and Guided Grad-CAM) applied to a correctly trained cat vs dog recognition model. (A) Cat example image. (B) Dog example image.

These visual tools create ‘visual explanations’ of decisions made by CNN-based models, making them more transparent and easier to understand (Selvaraju *et al*., 2017). In this article we will implement three of them, shown in Fig. 6 for the same cat vs dog recognition model, but now trained correctly: Grad-CAM, Guided Backpropagation and Guided Grad-CAM, the last being a combination of the first two (Gildenblat, 2017; Petsiuk, 2017; Nguyen, 2020). We have selected these tools, over others such as DeepLift (Shrikumar *et al*., 2017), Deconvolutional networks (Zeiler and Fergus, 2014), Integrated Gradients (Sundararajan *et al*., 2017) and Layer-wise Relevance Propagation (Binder *et al*., 2016) because, in addition to being some of the most common visual explainers (Buhrmester *et al*., 2021), they provide strong visual interpretability, which, after all, is the objective of Explainable AI approaches (Ali *et al*., 2023).

Grad-CAM generates a coarse spatial heat map by utilizing the gradients of a target concept (defined as such by the original Grad-CAM article but an object nonetheless, e.g. ‘dog’ or ‘cat’) as they flow into the final convolutional layer, highlighting key regions in the image that are important for predicting the concept (Selvaraju *et al*., 2017). As shown in Fig. 6, the emphasized areas (those that we should look at because they indicate the importance of that pattern for the classification) are the yellowish to reddish ones, with dark red areas being the most decisive in identifying the image’s class. In contrast, Guided Backpropagation modifies standard ReLU backpropagation to suppress negative gradients, allowing clearer visualization of the features of an image that contribute to network activation in the final layers (Springenberg *et al*., 2015).

Guided backpropagation, unlike Grad-CAM, provides a detailed view of how each pixel in the input contributes to the output of a network. However, this precision in fine details can make it difficult to interpret in terms of overall significance, which is what Grad-CAM excels at. Therefore, the best visual explainer is a combination of both techniques, the so-called Guided Grad-CAM (Selvaraju *et al*., 2017). This combined tool, as shown in Fig. 6, highlights the most relevant regions of the image and, in addition, emphasizes every important detail in those regions. It has been implemented with a Grad-CAM threshold of 0.75 to facilitate the visual interpretation (from 0 to 1, where 0 indicates insignificance and 1 represents a decisive influence in determining the image’s class). In this way, we will only highlight (in detail) the most significant areas indicated by Grad-CAM (the reddest ones).

### Dataset

The analysis, using the different visualization techniques mentioned above, has been performed on the 378 microscope images used in the original article to train, validate and test the phytolith identification model: 121 of *Avena sativa* (32.01 % of the total), 90 of *Hordeum spontaneum* (23.81 % of the total) and 167 of *Triticum boeoticum/dicoccoides* (44.18 % of the total). The images were taken on a Leica DM500 and a GXCAM-U3-5 digital camera with ToupeLite software. The images in this dataset are 2560 × 1922 pixels (px) in size and were previously cropped to 256 px for the development of the VGG19 model, the same cropped images (and therefore at the same resolution) to which the visual explainers will be applied. Using different image resolutions (different from the trained ones) would alter the results, but this is unnecessary since the extracted model’s information would remain the same if trained on the others.

This research uses all training, validation and testing data for the analysis, regardless of whether the data were used to train, improve or test the original DL algorithm. The visualization techniques will show us: 1) what the algorithm learnt when we apply them to the training and validation data, and 2) what features the model considers to classify the images into one class or another when we use the test data, the latter being a variation of the former as it is also based on what the algorithm learnt.

## RESULTS

**Fig. 7.**
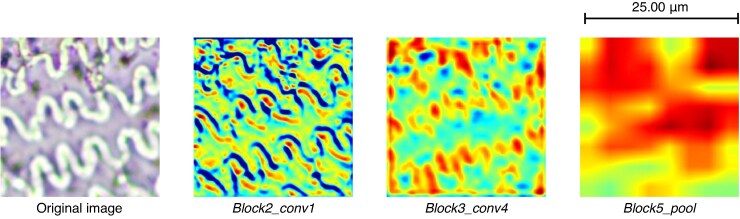
Grad-CAM applied to a *Triticum* 256-px crop example image for the following layers of the trained model: block2_conv1, block3_conv4 and block5_pool.

With the aim of making the black box more transparent, Grad-CAM also allows us to understand the learning process of the CNN model (Gunari *et al*., 2021). The algorithm focuses on the feature to be learnt thanks to the convolutions (Fukushima, 1980), first from basic elements (characteristics) such as edges and then from combinations of the elements (the multi-cell as a whole) learnt in previous layers (Lee *et al*., 2011), as observed in the progression through the different layers in Fig. 7.

**Fig. 8.**
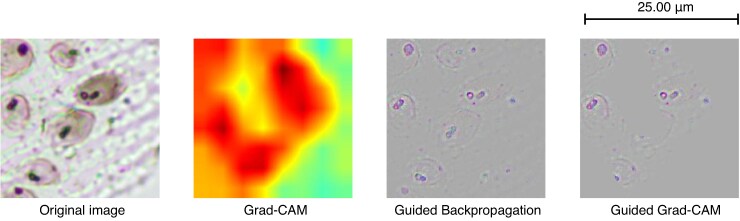
Grad-CAM, Guided Backpropagation and Guided Grad-CAM applied to an *Avena* 256-px crop example image.

**Fig. 9.**
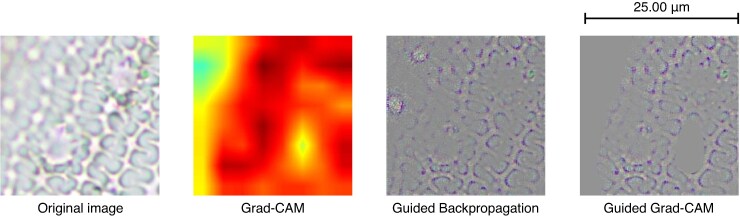
Grad-CAM, Guided Backpropagation and Guided Grad-CAM applied to a *Hordeum* 256-px crop example image.

**Fig. 10.**
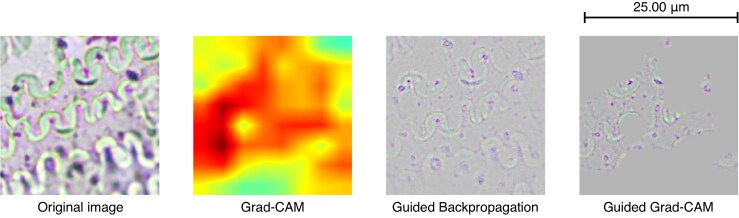
Grad-CAM, Guided Backpropagation and Guided Grad-CAM applied to a *Triticum* 256-px crop example image.

Below we present the results obtained when applying the mentioned visualization techniques to the phytolith images, using for this purpose an example of each of the three classes of the model: Grad-CAM, Guided Backpropagation and Guided Grad-CAM (Figs 8–10).

**Fig. 11.**
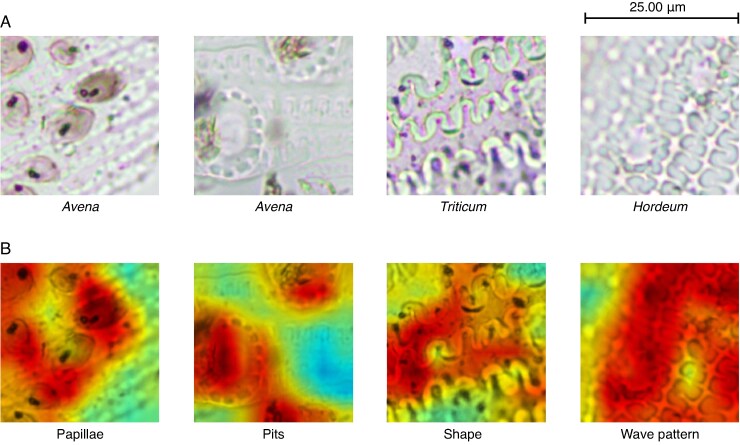
The different characteristics found and analysed after applying Guided Grad-CAM: papillae, pits, shape and wave pattern. The figure shows for each of the mentioned characteristics: (A) the original 256-px crop example image, and (B) Grad-CAM applied to the same image, which is shown over the original grey-scale image (the target concept is the yellowish to reddish area).

The analysis carried out using Guided Grad-CAM has allowed us to distinguish four characteristics or decision patterns when classifying the images of phytoliths in *Avena*, *Hordeum* and *Triticum*. These mentioned decision-makers are the papillae, pits, dendritic long-cell shape and wave pattern (Fig. 11). Likewise, we have been able to quantify which identifiable characteristics influence the classification for each of the three genera of phytoliths (Table 1).

**Table 1.** Influence of characteristics (as percentages) in phytolith classification for each of the three classes. The sum of percentages can be >100 % because phytolith images can have one or more patterns at a time as decision-makers for their classification.

	Papillae	Pits	Shape	Wave pattern
*Avena*	85.95	1.65	0.83	84.30
*Hordeum*	44.44	0.00	0.00	93.33
*Triticum*	10.18	1.20	38.32	95.21
Total	46.86	0.95	13.05	90.95

**Fig. 12.**
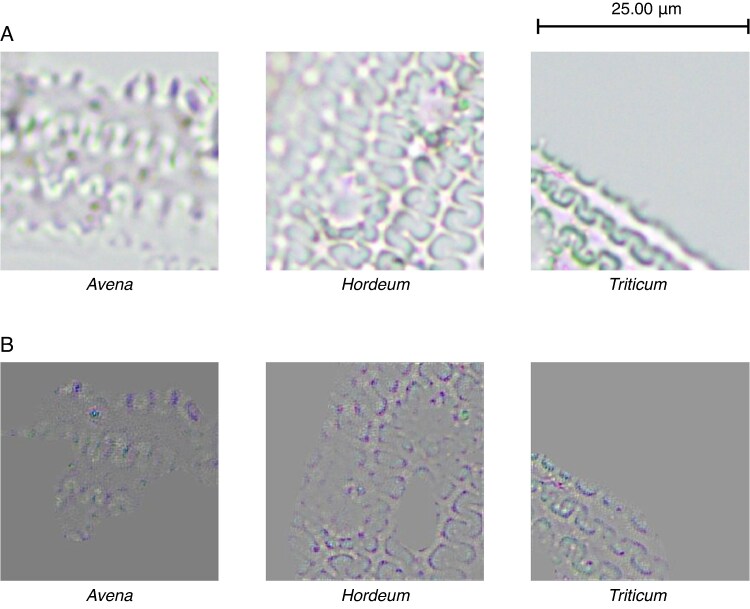
Wave pattern characteristic for each phytolith class. (A) Original 256-px crop example image. (B) Guided Grad-CAM applied to the same image.

**Fig. 13.**
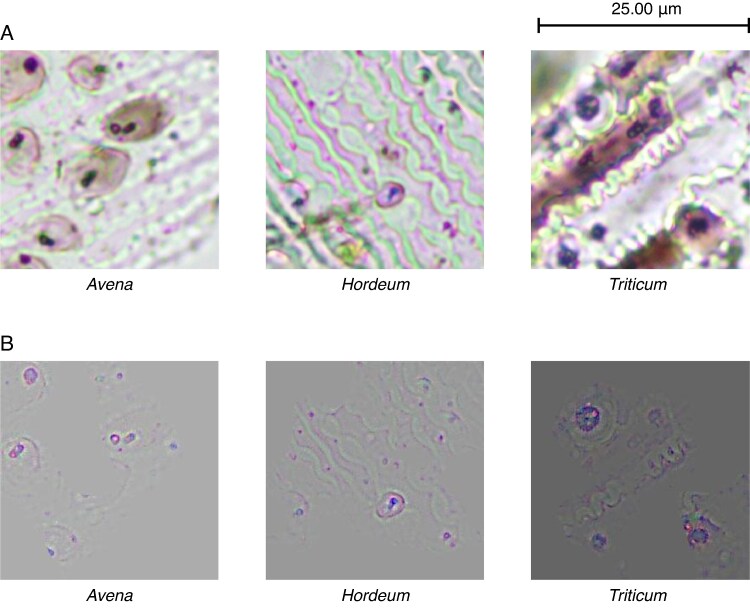
Papilla characteristics for each phytolith class. (A) Original 256-px crop example image. (B) Guided Grad-CAM applied to the same image.

**Fig. 14.**
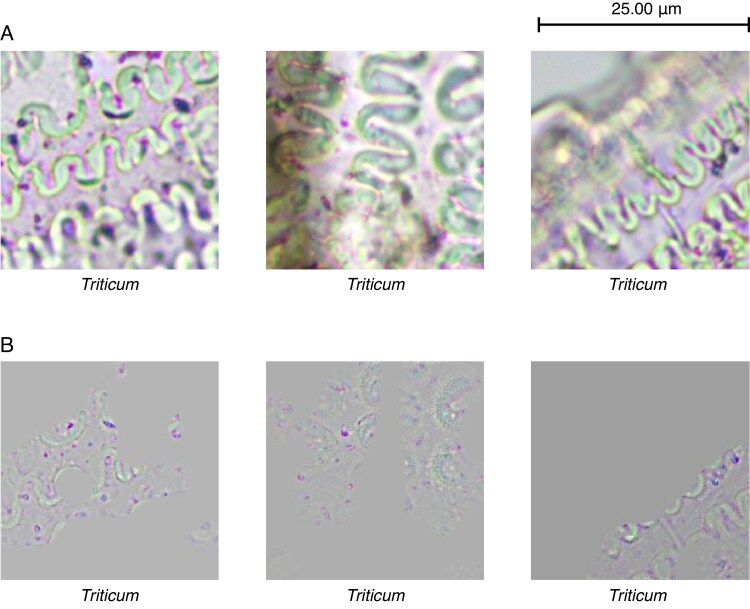
Dendritic long-cell phytolith shape characteristic for *Triticum* phytolith class. (A) Original 256-px crop example image. (B) Guided Grad-CAM applied to the same image.

From Table 1 it is evident that the wave pattern is key for the classification of phytoliths, with 91 % of the images being classified using this pattern (Fig. 12), individually or in conjunction with other characteristics mentioned. Likewise, the papillae are another of the features to be considered, mainly in *Avena* with 86 % and in *Hordeum* with 44 % (Fig. 13). On the other hand, it is worth mentioning that the dendritic long-cell phytolith shape is also key for the *Triticum* class, with 38 %, although to a lesser extent than the wave pattern (the negative space between the dendritic long cells), which accounts for 95 % (Fig. 14).

Regarding the papillae, there are only 241 images with them out of the total of 378 (63.76 %): 111 of *Avena*, 79 of *Hordeum* and 51 of *Triticum*. In this way, if we only analyse the images with papillae, we can observe that the papillae differentially inform the classification of certain genera: 93.69 % in *Avena*, 51.90 % in *Hordeum* and 33.33 % in *Triticum* (Fig. 13).

## DISCUSSION

To begin with, it is important to note that in the original article (Berganzo-Besga *et al*., 2022) two algorithms were developed, one for classification of multi-cell phytoliths at genus level and another at species level for the *Triticum* genus. In this research we have only focused on the first of the algorithms since, according to the original article, the model at the species level had a relatively small training dataset.

Regarding the model’s decision-makers, the wave pattern (Fig. 12) has been shown to be the main factor in classifying these three classes of phytoliths (Table 1). However, in *Avena* the papilla pattern stands out, with 86 % of images using them as decision-maker compared with 84 % of those using the wave pattern. Critically, considering only the images containing papillae, this characteristic is employed in 94 % of the images. Therefore, we can consider *Avena*’s papillae (Fig. 13) as their key characteristic with respect to the other two classes of phytoliths. Likewise, it is also worth mentioning that the dendritic long-cell phytolith shape pattern (Fig. 14) was a decisive factor for 38 % of the *Triticum* images; notably, this characteristic was not identified, independently of the wave pattern (negative space), by Rosen (1992).

**Fig. 15.**
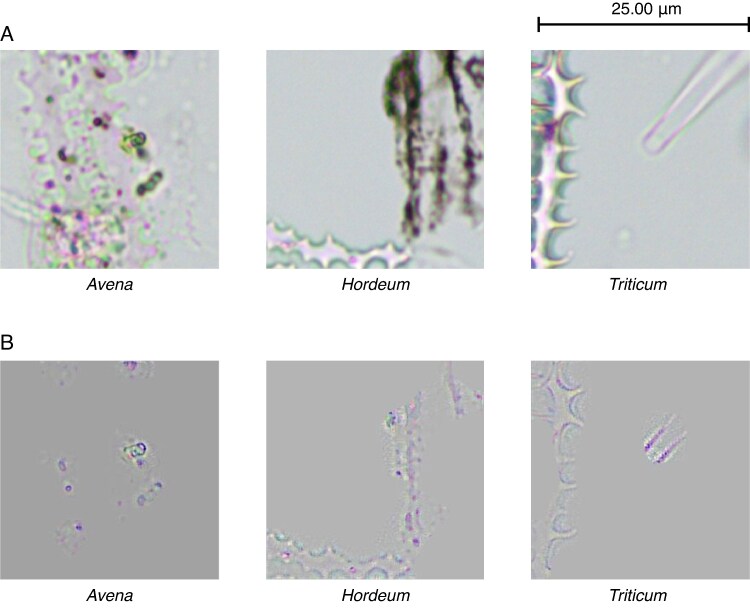
Different characteristics for each phytolith class. (A) Original 256-px crop example image. (B) Guided Grad-CAM applied to the same image. The second and third Guided Grad-CAM images also include wave pattern as decision-maker.

In addition to these key characteristics already discussed, in some images other potential characteristics are seemingly employed in the classification. However, these ‘features’ account for an insignificant number of classifications, probably due to some similarity to one of the images used during training and therefore not important, but should be considered for future retraining (Fig. 15). As mentioned, at this time it is not possible to identify a clear morphological characteristic that the algorithm is emphasizing in these anomalous situations.

Regarding the visualization tools, it should be noted that Guided Backpropagation requires the use of ReLU as the activation function and therefore can be less versatile, compared with Grad-CAM, on different architectures (Sundararajan *et al*., 2017). However, removing ReLU in Grad-CAM approaches may make the interpretation of the results less clear (Selvaraju *et al*., 2017).

Thus, the analysis of the microscope images using Guided Grad-CAM has confirmed the identifiable characteristics already known when classifying phytoliths (Rosen, 1992), such as reinforcing the importance of the wave pattern, but also demonstrated, unexpectedly, that different characteristics (papillae, dendritic long-cell shape and wave pattern) may be emphasized for different genera, and has allowed us to discover that dendritic long-cell shape, as a category on its own (i.e. rather than being an aspect of wave pattern), can also be characteristic.

This research has a clear focus on a series of computer vision best practices in archaeology. In recent years, in the field of computational archaeology attempts have been made not only to apply ML and DL but also to improve their application, such as reducing false positives by using multiple sources, algorithms and filters (Berganzo-Besga *et al*., 2021), and improving validation approaches in large-scale areas by taking into account low-density scenarios (Berganzo-Besga *et al*., 2023). As we have seen, results can also be masked in image classification contexts, as in the black box case we have analysed in this article.

Therefore, both to ensure good learning by the algorithm and to find the hidden patterns that AI might reveal, we should integrate these visual explainers into standard practice.

## Data Availability

The code to apply Guided Grad-CAM (Gildenblat, 2017; Petsiuk, 2017; Nguyen, 2020 ), adapted to the identification of phytoliths using the trained VGG19 model (Berganzo-Besga *et al*., 2022), can be found in a Google Colab notebook at the following Github repository link: https://github.com/iberganzo/ArchaeolGradCAM.
